# Salinity Measurement with a Plasmonic Sensor Based on Doubly Deposited Tapered Optical Fibers

**DOI:** 10.3390/s24154957

**Published:** 2024-07-31

**Authors:** María-Cruz Navarrete, Natalia Díaz-Herrera, Agustín González-Cano

**Affiliations:** 1 Optics Department, Faculty of Physics, University Complutense of Madrid, Ciudad Universitaria s/n, 28040 Madrid, Spain; mcnavarr@ucm.es; 2 Optics Department, Faculty of Optics and Optometry, University Complutense of Madrid, Arcos de Jalón 118, 28037 Madrid, Spain; agugonza@ucm.es

**Keywords:** surface plasmon resonance, fiber optic sensors, tapered optical fibers, salinity, seawater

## Abstract

Salinity is a very important parameter from an environmental perspective, and therefore, efficient and accurate systems are required for marine environmental monitoring and productive industries. A plasmonic sensor based on doubly deposited tapered optical fibers (DLUWTs—double-layer uniform-waist tapers) for the measurement of salinity is presented. The physical principle of the sensor, as well as its structure, is discussed, and its performance is experimentally demonstrated, obtaining very good sensitivities. The possibility of shifting towards higher wavelength measuring ranges associated with DLUWTs is also exploited. At the same time, we have considered the necessity of an extensive characterization of the behavior of the refractive index of salty water, both with variations in temperature and the composition of the salts dissolved. This is important due to the somehow changing reality of salinity measurements and the possibility of establishing new approaches for the determination of absolute salinity as opposed to practical salinity based on electrical conductivity measurements. The results obtained, which show high sensitivity and a good performance in general without the need for the use of semi-empirical algorithms, permit, in our opinion, an advance in the tendency towards refractometric determination of salinity with optical sensors apt for in situ, real-time, accurate measurements in realistic measuring conditions.

## 1. Introduction and State-of-the-Art

Salinity is the most important parameter from the environmental point of view. It strongly affects life in the marine environment, and a correct understanding of the dynamics of its distribution is crucial for climate change studies. It is also crucial for productive industries and for marine environment monitoring [1,2,3]. This is why, over the years, many methods have been proposed to measure it. In recent times, the pressure to have real-time, in situ operating systems and platforms that can provide the required accuracy has increased. For that reason, many salinity sensors have been proposed in the literature, and several different methods for measuring salinity are now considered [4].

However, the fact is that, as we will discuss in more detail in the next sections, nowadays, there remains some confusion in the mere definition of “salinity” that obscures in some way the discussion on which is the optimal way of approaching its measurement. The intuitive concept that relates “salinity” with the concentration of dissolved matter in seawater is not always directly applicable to realistic measurement conditions, and the use of so-called “practical” scales based on indirect measures has been common for decades, up to the point of “merging” the two concepts, namely, the “real” salinity and some “practical” salinity measured, most commonly, via electrical conductivity.

This, as we will see, is unsatisfactory in several ways, and for that reason, in at least the last twenty-five years, there has been an increasing interest in achieving new methods of measuring absolute salinity not based on the traditional approach. In the literature, we can find a significant amount of proposals aimed at shifting the perspective, privileging the use of optical methods, and not considering any more electrical conductivity but the refractive index as the direct parameter to be used [5,6,7,8].

The potential advantages of the optical approach will be discussed in detail in Section 2, but it is important to note here that our contribution to the field goes in that direction. If we provide better and more reliable methods of measuring refractive index and salinity, the transition to perhaps a new standard that could provide better knowledge of absolute, not only practical, salinity.

Our group was, we could say, one of the pioneering groups to develop optical fiber-based plasmonic refractometers to measure salinity, having achieved even in situ measurements in oceanographic campaigns [9,10]. Since that time, obviously, many other contributions have been made. Still, the fact is that the desired paradigm shift is not already completed because of the inertia inherent in any established measuring scheme and the corresponding difficulty of changing habits and operating systems, and the relative importance of conductivity-based measurements of salinity is still huge, even when the optical approach offers advantages.

Once we decide to measure the refractive index to obtain salinity from it, the existence of very practical, versatile fiber refractometers facilitates their use in measuring platforms or campaigns in the open sea or in coastal facilities. The need for in situ, real-time monitoring with high accuracy levels is increasing and the fact that fibers can be easily operated at long distances and can, therefore, be used to reach any difficult places without the need for sophisticated arrangements is a key point and places fiber sensors as the best candidates for this change of perspective [11,12,13].

Within the field of optical fiber-based refractometers, it is obvious that plasmonic devices (based on so-called surface plasmon resonance, SPR) offer the best performance in terms of sensitivity. The use of plasmonic devices for salinity measurement is not, of course, novel. We already used a plasmonic D-shaped fiber device in 1999 [9], and many other interesting proposals in that direction have been present in the literature in recent years [14,15,16,17,18]. However, doubly deposited uniform-waist tapered optical fibers (DLUWTs), which are amongst the most versatile and sensitive plasmonic refractometers and that have repeatedly shown good performance in varied wavelength ranges [19], have not been used until now to thoroughly investigate the question of the optical measurement of salinity. The main reason for publishing this paper is to show that, with this technique, we can exploit the potential of plasmonic refractometers in an interesting way because of the positive characteristics of DLUWTs. Among others, DLUWTs show high sensitivity, simplicity in design and the fact that they can operate in a variety of spectral ranges. Additionally, using DLUWTs instead of the D-shaped fibers simplifies the setup because the dependence on polarization is greatly reduced. Also, in this paper, we discuss in detail the mere question of how optical measurements of salinity could be the best alternative to the current state of affairs.

The structure of the paper reflects our intentions. First, a reflection based on the historical perspective addresses the different ways of measuring salinity and their potential advantages and disadvantages is made in Section 2. Then, in Section 3, an extensive experimental characterization and comparison of the behavior of the two main parameters used for indirect measurements of salinity, namely electrical conductivity and refractive index, is performed, including an assessment of the dependencies of these measurements with temperature and the composition of the “salts” dissolved in water. Section 4 summarizes the concept, fabrication, and experimental performance of DLUWTs, devices that we apply to determine salinity. The discussion on the performance of DLUWTs as salinity measuring devices is made in Section 5, where we show how they can advantageously be used in real-time in situ measuring conditions, such as in coastal facilities or marine platforms. Finally, in Section 6, some conclusions are presented, and some future developments are suggested.

## 2. The Question of the Measurement of Salinity

In general, water masses are divided into fresh and salty, and for the most part, saltwater is related to the sea. The products dissolved in seawater are varied, with the chlorides being predominant. Salinity is then a measure of the relative amount of these products. In general terms, salts represent about 3.5% of the total mass of seawater, with chlorine and sodium (about 55% and 30%, respectively) being the most abundant components of this “salty” matter. Sulfur, calcium, potassium, magnesium and other elements are present in smaller quantities. Chlorides—NaCl and KCl—are the most prominent salts in seawater, but not by any means the only ones [20].

As we have mentioned, the questions concerning the definition and the measuring technique for salinity are only partially solved. A first and intuitive concept of salinity, apart from the immediate idea that salinity is a measure of what we qualitatively identify as the “saltiness” of water, i.e., the difference between freshwater as in rivers and saltwater as in the oceans, could be to relate salinity with a measure of concentration. In that sense, we can consider a given mass of water, and the relationship between the masses of the dissolved products and the mass of the dissolvent would be a measure of salinity. This has become what is called “absolute salinity” and is the parameter that appears in the Thermodynamic Equation of Seawater, TEOS-10, established in 2010 [6,20,21]. It is expressed in g·kg^−1^, or equivalently in ‰ or parts-per-thousand (ppt).

There are some problems associated with this simple equivalence between salinity as a global parameter and concentration of dissolved compounds. First, it is not always easy to measure it in field conditions since dissolved matter in seawater is a complicated mixture. In the laboratory, chemical procedures to determine it can be applied, but this is not efficient when we try to determine in situ values, for instance, in the open sea, the salinity of a given sample, and even less so if we are thinking of an environmental monitoring platform.

So, over the years, we have been opting for indirect measurements to determine salinity via an intermediate parameter. Historically, the best results were achieved when linking salinity with the variation of electrical conductivity. The technical possibilities of accurately measuring conductivity even for non-lab environments were promising some decades ago, so a decision was made that still essentially holds: to decree an “equivalence” between the scales of salinity and conductivity. The physics and chemistry behind this association rely on the fact that some of the salts present in seawater are polar, so, for instance, the amount of chloride ions, Cl^−^ is directly responsible for a change in conductivity. As chlorides are the most abundant of salts in the marine environment, it was an easy step (another “equivalence”) to equate salinity with chlorinity. Accordingly, a new standard was established in 1978 known as PSS78 [22].

Measuring conductivity proved to be simple. The unit of conductivity is the Siemens, equivalent to the inverse of an ohm, i.e., to the quotient between ampere and volt. However, as we can see, a chain of associations is fabricated that is somehow arbitrary: salinity as concentration is the same as salinity as the means by which conductivity changes and salinity is then equivalent to chlorinity. The fact that this is not a sufficiently sound way of proceeding is shown in need to define a new, not-SI unit—the so-called “practical salinity unit (psu)”, whose use is no longer advised. This corresponds to a proportion between the response of the conductimeter to the problem sample and the response to a standard solution. There is, then, the need for an official standard for “seawater” (provided by IAPSO and manufactured by only one company, Ocean Scientific International). If we have this standard, for instance, the one that corresponds to the value of salinity (S) equal to 35 “psu”, assumed the “normal” salinity for seawater, contains 32.4356 g of KCl per kilogram of water at 15 °C.

However, there is another tacit assumption that may be problematic. The idea is that one has the same composition of “salt” in all the oceans of the planet; that is to say, we can have different values for the salinity measured by the presence of chloride ions and the proportion of these ions with respect to the total of products dissolved all around the planet is considered as kept the same. This is called the principle of constant proportions, and it is what justifies the possibility of using one single property scalable for the measurement of a rather complex parameter, such as the concentration of dissolved matter (with different compositions) in water [23]. This has been proven wrong. There are significant deviations between the composition of “salt” in different regions, so we can have the same reading by the “salinometer” (i.e., the conductimeter) and have different concentrations of dissolved matter. This has been called the “anomaly of absolute salinity”, and we have tables and algorithms to perform a correction from the values obtained with commercial salinometers [6].

Finally, electrical conductivity is known to be significantly dependent on temperature and, in a lesser way, on pressure, so it has been common to integrate not only one but three sensors in what is called a CTD probe (conductivity–temperature–depth). An algorithm is used to combine the readings of these three parameters to obtain a final measure of salinity.

Although the scientific community is conscious of the limitations of this approach, and this has been clearly shown when a new standard was adopted in 2010, TEOS-10, in which the use of absolute, rather than practical, salinity is prescribed, the fact is that conductimeters are still today the most widely used instruments to measure salinity both in the field and in the laboratory. However, we can admit that optical measurements would be preferable because the refractive index of water is more directly related to the concentration of dissolved salts than conductivity, and it also responds to nonpolar compounds. Furthermore, and this is very important, the refractive index correlates with density better than conductivity and can be used to determine absolute, not practical salinity, so it would be wise to shift the interest in salinity measurements from the electrical to the optical field and consider the possibility, of a paradigm shift and a change in the standards [24,25].

It is true that some years ago, electrical sensors were more developed than optical sensors, but this is no longer the case. In particular, the resolutions, sensitivities and accuracies reachable today in refractometry are among the best one can obtain for any optical measurements when using surface plasmon resonance, as it has repeatedly proven over the years in the literature [26]. In that sense, it could be conceivable to establish a new “equivalence” between salinity and refractive index, which would substitute the existing one with the electrical conductivity. The refractive index responds to the variation of the relative concentration of the compounds, so the problem of the anomaly of absolute salinity could be treatable with the necessary calibrations. At last, given the fact that SPR sensors are among the best possible options for chemical sensing, it could be feasible to even improve the performance of the sensors by the addition of some reagents that could tune the response of the plasmonic devices to the target analytes desired [27]. Although optical SPR salinity sensors have been proposed in the literature and tested in the field for years, as has been reported in the literature and mentioned in the previous section, this paradigm shift is still not occurring. We will try to contribute to it by presenting a versatile, high-performance fiber-optic device suitable for use in realistic, even hostile conditions, which we will depict in the following sections.

## 3. Dissolved Salt Concentration and Its Relation to Salinity, Temperature and Refractive Index

First, we are going to extensively characterize the dependencies of salinity with the two mainly measured parameters to determine it, namely electrical conductivity and the refractive index. We will also take into account the dependency on temperature and the fact that the exact composition of the dissolved products in seawater affects the measurement.

We have used three different kinds of salt for the study: Potassium Chloride (KCl, from QP Chemicals, Valencia, Spain) as it is used as the common reference for conductivity measurements of salinity; Sodium Chloride (NaCl, from commercial coarse refined sea salt) as it is the most common component of seawater, and so-called aquarium salt (Pro-Reef Sea Salt for aquariums by Tropic Marine, Wartenberg, Germany, [28]), which in principle is manufactured to better simulate the real components of seawater.

In terms of measuring instruments, we used a commercial salinometer (model 162, ATI Orion, Boston, MA, USA) for the electrical conductivity and an Abbe refractometer (NAR-3T, ATAGO Co., Ltd., Tokio, Japan) for the refractive index. Temperature was measured with the temperature sensor incorporated in the salinometer mentioned before.

### 3.1. Electrical Conductivity Measurements of Salinity vs. Salt Concentration

We have said that nowadays, salinometers based on electrical conductivity are by far the most common instruments for determining salinity. However, the relationship between the concentration of salts in water and conductivity is not that simple. So, we begin by experimentally determining this relationship for a variety of salt concentrations and compositions. This is important for us since we are using the salinometer as a reference measurement to compare with our refractive measurements.

**Figure 1:**
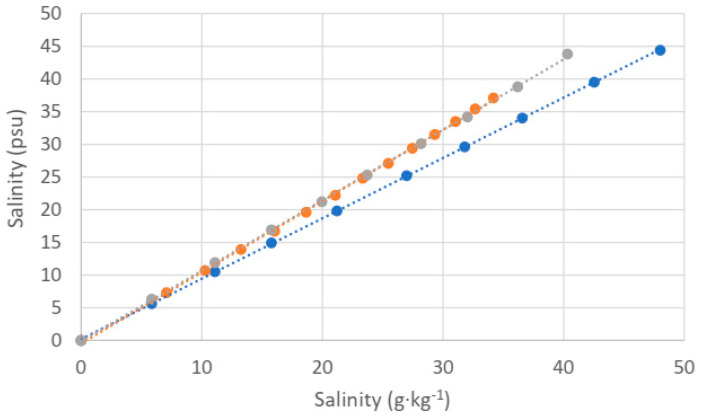
Measured salinity through conductivity related to the sample concentration (g·kg^−1^): KCl (orange; linear fit: y = 1.0895x − 0.4784, R^2^ = 0.9995), NaCl (grey; linear fit: y = 1.0773x − 0.1111, R^2^ = 0.9998), Tropic Marine (blue; linear fit: y = 0.9237x + 0.191, R^2^ = 0.99996).

The sample consists of a mixture of deionized water and the salt chosen for each measurement series, and we vary the concentration in a defined way by adding the appropriate amount of salt to 1 L of water. We have used a precision balance to weigh the amount of salt (Analytical Balance PW 254, Adam Equipment Co., Ltd., Milton Keynes, UK). We show the obtained experimental results in Figure 1.

As expected, the linearity is good in all three cases. However, the three curves corresponding to the three different types of salt do not coincide. When dealing with chlorides (KCl and NaCl), there is a good overlap, which is expected since electrical conductivity is strongly related to chlorinity. The salinometer does not distinguish between these two different compounds. However, the curve corresponding to aquarium salt deviates from the others because, although chlorides (specifically NaCl) can still be the dominant components, we can find many other types of dissolved products, making the relative amount of Cl^−^ proportionally smaller. As we have discussed, this is an undesired feature of electrical conductivity-based measurements of salinity, and it is related to the so-called anomaly of absolute salinity. We see that if we take aquarium salt as the most approximate one to a realistic salt content in seawater, the error in the reading could be considerable, requiring a larger amount of aquarium salt to obtain any figure of practical salinity, for instance, 30 or 35 psu. For those values, we would require at least several additional grams of dissolved matter.

### 3.2. Electrical Conductivity Measurements of Salinity vs. Temperature

Electrical conductivity is obviously dependent on temperature. We characterize this effect in the measures made with the salinometer here. To do that, a sample with a fixed salt concentration is monitored with the salinometer while its temperature is gradually increased. Any variation in the response must then be due, in principle, solely to the temperature since the ions in play do not change, and the salinity itself should not change either. This is widely known, and commercial salinometers include a parallel temperature sensor and perform by default a numerical correction through the use of an algorithm to take into account this potential effect, adjusting the response so the finally provided value is that which would correspond to a temperature of 20 °C. This implies that if that correction is properly made, and the components dissolved in water have not varied, the salinity would not be varied, and the readings of the salinometer should not change.

The algorithm employed is usually quite opaque in commercial devices, and the specifications provided by the manufacturer, in our case, establish an uncertainty of the order of 0.5% (not negligible at all) due to “temperature compensation”.

We checked how it worked with our salinometer for two samples, with KCl and aquarium salt, as well as for different values of the corresponding salt concentration. The desired concentrations of the several samples were achieved by the method detailed in Section 3.1. The salinometer was adjusted for every measure of the salinity to the value for a temperature of 20 °C.

**Figure 2:**
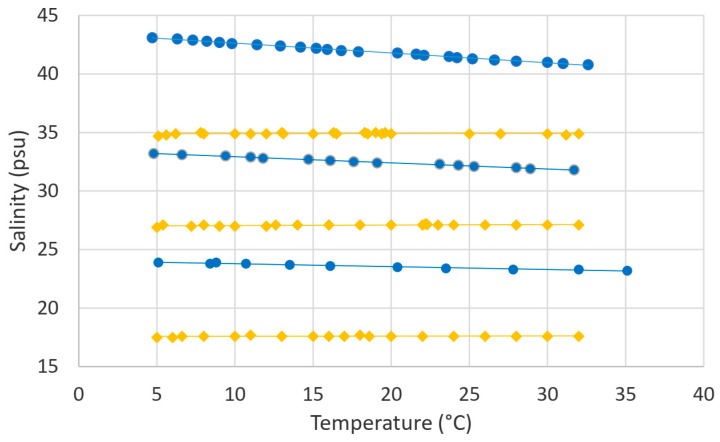
Salinity was measured with a salinometer as a function of temperature for a given salt concentration (19.4, 28.9 and 38.2 g·kg^−1^) in mixtures of KCl (blue) and aquarium salt (yellow).

The results are shown in Figure 2.

As can be seen, the behavior is different for the two types of salt. For aquarium samples, we obtained a horizontal line indicating there was no drift due to temperature variations once the algorithm was applied. On the other hand, the salinity provided by the salinometer for KCl samples decreased when the temperature increased. This variation indicates that the salinometer algorithm for adjusting the value to 20 °C in the case of this salt is not very suitable since there is some variation in the measurement due to temperature drift, so the response of the salinometer is temperature-dependent for KCl, although we are using an adjusting algorithm. This is not the case for aquarium salt, where the measurement remains almost constant over the whole range, so the salinometer adjustment works better with aquarium salt than with KCl.

The fact that the drift in salinity measurements with temperature, even when “compensation” is applied, depends on the exact composition of the salt is significant, but otherwise rather not unexpected, since conductivity is already different for different types of salt. From the linear fitting for each concentration of KCl, we can see that it varies more for higher concentrations, while the slope is steeper: −0.08 psu/°C for 38.2 g·kg^−1^ (linear fit: y = −0.0839x + 43.471, R^2^ = 0.9968); −0.05 psu/°C for 28.9 g·kg^−1^ (linear fit: y = −0.0526x + 33.455, R^2^ = 0.9952) and −0.02 psu/°C for 19.4 g·kg^−1^ (linear fit: y = −0.0247x + 24.035, R^2^ = 0.9692). In the worst case, the highest concentration at 30 °C varies around 2.5 psu (0.08 psu/°C), which is a significant variation.

This means that the use of a correction algorithm and a parallel temperature measurement is mandatory for electrical conductivity-based measurements of salinity. If the algorithm is not used, the temperature dependence of electrical conductivity is important (as much as ten times the dependence of refractive index with temperature [6]). Therefore, we are obliged to rely on an internal mechanism of “temperature compensation”, which is not usually made explicit by the manufacturer and, in some cases, is impossible to disconnect. When the algorithm is used, we still have undesired variations of the salinity readings that compromise the accuracy of the whole measurement scheme.

### 3.3. Refractive Index vs. Salt Concentration and Temperature

We are presenting a device that can measure salinity by determining refractive index, so the next logical step would be to characterize the refractometric behavior of samples with different amounts of salt dissolved in water. For the refractive index, the dependency on temperature is not as high [29,30,31], being in the order of 1·10^−4^ RIU/°C for water and in the order of 4·10^−4^ RIU/°C for other liquids; however, we will, in any case, take it into account [32].

The most important point is to establish the experimental relationship between the salt concentration in a solution and its refractive index. To do this, we have fabricated several mixtures of deionized water and KCl. The refractive index of each sample is measured with the Abbe refractometer.

**Figure 3:**
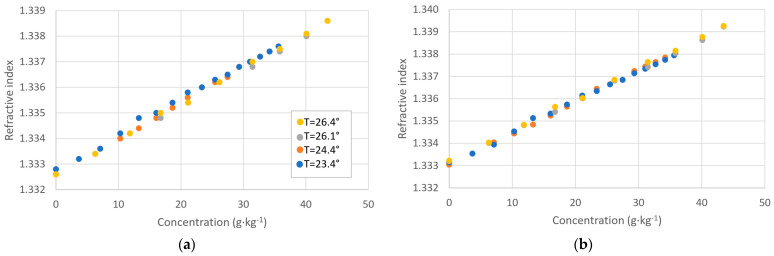
(**a**) Refractive index as a function of the sample concentrations for KCl mixtures in several tests at different measurement temperatures; (**b**) Same measurements corrected at a T = 20 °C.

The results are shown in Figure 3a. We can see that the behavior is linear and the slope is of the order of 1.4·10^−4^ RIU/(g·kg^−1^), which implies that with the achievable resolutions, for instance, with plasmonic devices, we can measure salinity with a high degree of accuracy.

In the graphic on the left in Figure 3a, we show the results for the temperatures at which the measurements were taken for each cycle. A small variation of temperature in the lab is unavoidable, so we corrected the readings, obtaining the equivalent values for a reference temperature of 20 °C by introducing a factor of 1·10^−4^ RIU/°C, which was expected in that the index of water varies with temperature. This is shown in the graphic on the right, Figure 3b. When corrected in terms of temperature, the variability between measurements is reduced. This temperature correction will be taken into account from now on for the refractive index measurements.

**Figure 4:**
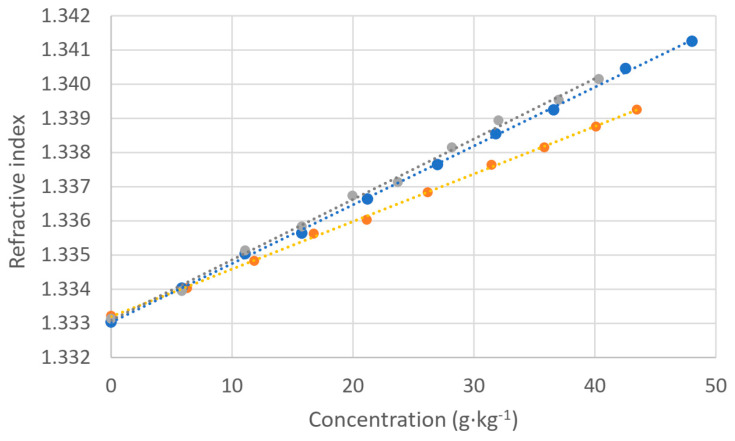
Refractive index as a function of the sample salt concentration, corrected at 20 °C, for three different salts: KCl (orange; linear fit: y = 0.00014x + 1.33320, R^2^ = 0.9992), NaCl (grey; linear fit: y = 0.00018x + 1.33309, R^2^ = 0.9974), and aquarium salt (blue; linear fit: y = 0.00017x + 1.33303, R^2^ = 0.9994).

We are also interested in exploring the variation of the refractive index when varying the salt concentration for different types of salts. The refractive index of the samples with known salt concentrations is measured with the Abbe refractometer and corrected to obtain the equivalent at a temperature of 20 °C so that the effect is due only to the type of salt. The results are shown in Figure 4.

It can be seen that the curves for NaCl and aquarium salt are very similar, as expected, since sodium chloride is the main component of the sea salt. The curve for KCl differs from the previous ones, and the refractive index for the same concentration of salts is lower for KCl samples. The behavior here is different from that of electrical conductivity because the refractive index does not depend on chlorinity.

We have obtained a linear behavior again in all cases, and from the linear fitting, we can see that the slopes of the curves are: 1.4·10^−4^ RIU/(g·kg^−1^) for KCl; 1.8·10^−4^ RIU/(g·kg^−1^) for NaCl and 1.7·10^−4^ RIU/(g·kg^−1^) for aquarium salt.

### 3.4. Refractive Index vs. Electrical Conductivity in the Measurement of Salinity

**Figure 5:**
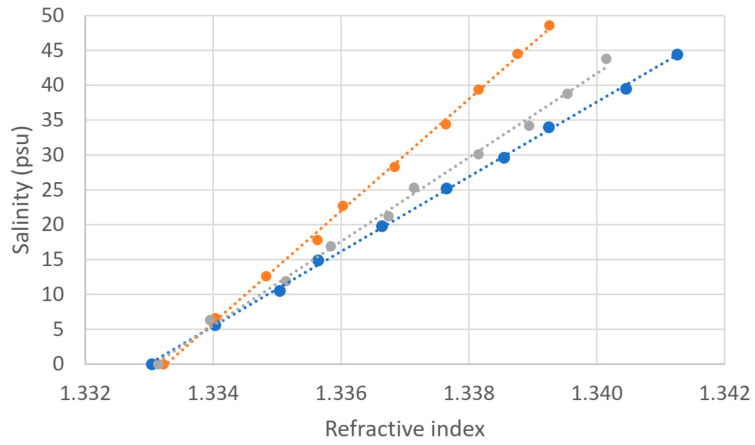
Salinity measured by a salinometer as a function of the refractive index obtained by an Abbe refractometer corrected at 20 °C: KCl (orange; linear fit: y = 8026.4x − 10701, R^2^ = 0.9987), NaCl (grey; linear fit: y = 6024.2x − 8030.8, R^2^ = 0.9968), and aquarium salt (blue; linear fit: y = 5362.3x − 7147.9, R^2^ = 0.9993).

Our final step is to compare the responses of the salinometer and the refractometer for a given concentration of salt. For this, we prepared the measured solution, starting with a volume of 300 mL of deionized water and adding small volumes of salty solutions with the three types of salt. The concentrations of the salty solutions for each salt were 56.6 g·kg^−1^. We adjusted the values to a reference temperature of 20 °C. The results are shown in Figure 5.

As can be seen, the linearity is quite good in all cases, but again, the behavior depends on the type of salt used. The slopes of the curve fitting are the following: 8026.4 psu/RIU for KCl, 6024.2 psu/RIU for NaCl, and 5362.3 psu/RIU for aquarium salt. We can see that the variation is important and that, for the same value of refractive index, we can have different practical salinities. If we agree that the refractive index is more directly related to the concentration of dissolved salts than electrical conductivity, which is strongly dependent on Cl^−^ ions, this could be significant in real measurements. Again, the curves of NaCl and aquarium salt are nearer than that of KCl because NaCl is higher in aquarium salt than KCl. The effect is more discernible here than if we only account for the electrical conductivity-based measurements of salinity. This could be an interesting feature if the discrimination of the dissolved products is one of the goals of the measurements. If we, for instance, start with an initial calibration, we can determine which curve is to be applied, thus obtaining a more accurate result.

With all these measurements, we can see a difference between the use of conductimeters and refractometers, and this difference can be significant in several ways since the exact composition of salt in seawater and temperature are not negligible parameters.

## 4. SPR Refractometer Based on DLUWTs for Salinity Measurements

Once we admit that a refractometric measurement of salinity is desirable, the most promising choice of refractometer is based on plasmonic fiber-optic devices. In general, plasmonic devices rely on the resonance mechanism established between a guided wave and a plasma wave occurring in a superposed metallic layer. The resonance matching conditions are very precise—this allows us to measure the refractive index of the external medium, which is the value of the wavelength where the resonance is produced and is variable with that refractive index. Several possible ways of achieving this resonant structure have been proposed, and in our case, we use evanescent-field fiber sensors since the evanescent wave possesses the right wave vector to eventually excite plasma waves on deposited layers.

We used D-shaped fibers with a double metal-dielectric deposit several years ago. The results were good, and the device was tested and demonstrated in campaigns in different seas in real operating conditions [10]. However, one undesirable feature of this kind of device, due to the asymmetry of the deposit, is its strong dependence on polarization, so the presence of polarization-controlling elements was mandatory [9].

This dependence on polarization is strongly decreased, even to the point of no longer requiring any additional polarization control, when we use the cylindrically symmetric doubly deposited uniform-waist tapered fibers (DLUWTs) as shown in previous work [33].

Another major advantage of DLUWTs is their double deposit, e.g., the addition of a second dielectric layer to the metallic layer where plasmon is excited, is their versatility, and the simplicity with which we can modify the working spectral region by just changing the thicknesses or the materials employed [34,35,36,37].

We have been working with this kind of structure for years, and we have applied them to different types of measurements, sometimes combining the transducer with other materials to couple the response as refractometers to any measurement or analyte we analyze [19]. However, the direct, straightforward approach to salinity refractometric measurements via DLUWTs has not been made until now. We show how we can take advantage of the very good behavior of these transducers to improve the way salinity refractive sensors work.

### 4.1. Device Fabrication and Characterization

We have depicted in detail both the fabrication scheme for the devices and their characterization process [19]. We summarize here those matters for the sake of completeness.

As mentioned, the sensor is based on a fiber taper with a double-layer deposition. The tapered fibers are fabricated by the so-called traveling burner technique [38]. We have fabricated tapers for working both in the visible (Newport FSA single-mode fiber, with a 30-micron waist, which has demonstrated a good relationship between sensitivity and robustness) and near-IR (Newport F-SMF-28 single-mode fiber, with a 335-micronwaist) in order to compare their performances. Two layers of aluminum, Al, and titanium dioxide, TiO_2_, respectively, were deposited onto the tapered zone by the CVD method. The thickness of the layers is designed so that the plasmon resonance is in the desired working wavelength range for refractive indices of the external medium corresponding to aqueous media. This design process has been optimized in the past and has been described in the literature. In this work, the thickness of the Al and TiO_2_ layers were 8 nm and 25 nm, respectively, to provide resonances in the visible range, with which we obtained a plasmon resonance at 535 nm for deionized water as an external medium. The thickness of the Al and TiO_2_ layers to operate in the near IR range were 19 nm and 99 nm, and the plasmon resonance was obtained at 1600 nm in the same condition.

**Figure 6:**
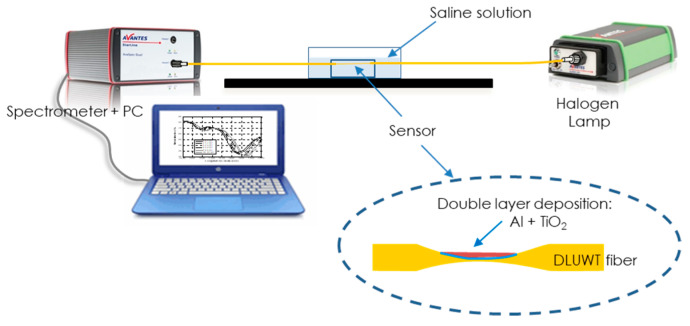
Experimental setup for the sensor characterization.

To characterize the behavior of these SPR sensors, the spectral transmittance was measured when the refractive index of the external medium changed. When the matching conditions were fulfilled, the plasmon excitation manifested as a minimum in the measured spectral transmittance in the so-called “plasmon curve” [39]. The experimental setup (see Figure 6) was the usual one: a halogen light source (AvaLight-Hal, Avantes BV, Apeldoorn, The Netherlands) connected to the fiber on which the sensor is fabricated, the sensor placed in a cuvette with a magnetic stirrer to homogenize the mixture, the light passing through the sensing area collected by a PC-controlled spectrometer. Depending on the working wavelength range, a spectrometer was used for the visible region (AvaSpec 2048, Avantes BV, Apeldoorn, The Netherlands) or the near-IR (AvaSpec-NIR256-1.7, Avantes BV, Apeldoorn, The Netherlands).

The usual method for characterizing the behavior of these sensors is carried out. We generated variations of the refractive index of the external medium while recording the spectral transmittance to determine the displacement of the minimum corresponding to the plasmon resonance. The sensor calibration curve is thus obtained. This was made by adding ethylene glycol to a deionized water sample, as it allowed a wide range of refractive indices of the external medium to be covered. The refractive index of each sample was measured with the Abbe refractometer.

**Figure 7:**
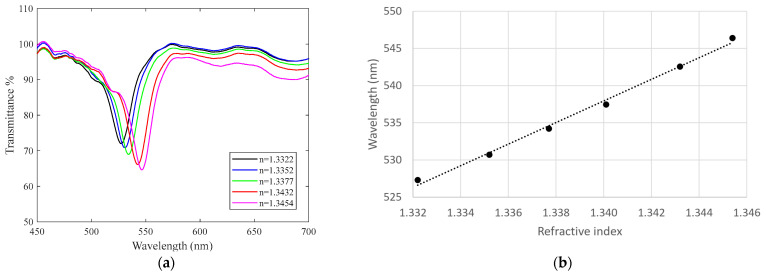
(**a**) Spectral transmittance variation due to outer refractive index changes. (**b**) Dependence on the position of the wavelength associated with the plasmon resonance with outer refractive index (linear fit: y = 1451.9x − 1407.6, R^2^ = 0.9943).

As mentioned, the plasmon excitation results in a minimum in the spectral transmittance. So, we plotted the position of the minimum for each refractive index of the external medium to obtain the curve. Figure 7 shows the spectral transmittance as the external medium varies for a sensor in the visible range to show a representative behavior of DLUWT-based fiber refractometers.

It can be seen that, as it is well known, as the refractive index increased, the minimum associated with the plasmon resonance shifted to longer wavelengths. By performing a linear fit of the minima position, the sensitivity of the sensor to variations in the refractive index can be obtained. In this case, Figure 7b is of the order of 1452 nm/RIU.

### 4.2. Influence of Temperature on the Resonance Minima

Since temperature changes the value of the refractive index and will therefore affect the position of the resonance minima, it is important to evaluate the effect of temperature variations on the position of these minima.

For this purpose, the sensor was placed in a mixture of deionized water and aquarium salt with a salinity of 30 psu measured with the salinometer, and, without changing the composition of the external medium, the temperature increased from 5 to 40 °C.

**Figure 8:**
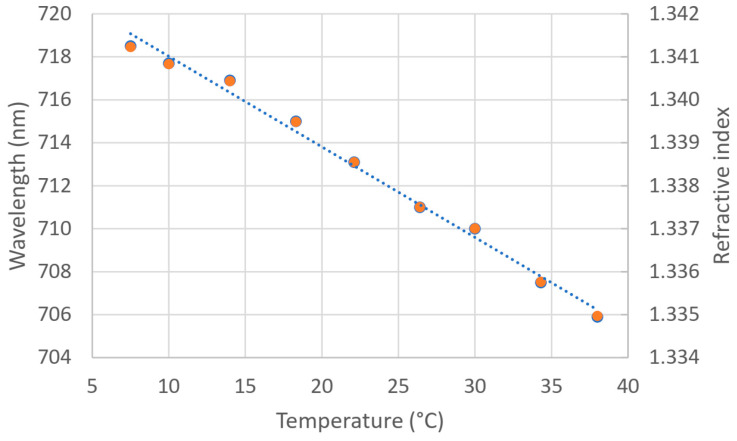
Effect of temperature variations in the position of the plasmon minima with a fixed external medium. Main axis: experimental data (linear fit: y = −0.4213x + 722.24, R^2^ = 0.9917); secondary axis: corresponding refractive index of the external medium by using the calibration curve (linear fit: y = −0.00021x + 1.34308, R^2^ = 0.9917).

A different sensor was used, but it was still in the visible range. The results are shown in Figure 8. The experimental values are plotted on the main axis (blue), almost covered by the corresponding refractive index values in each case, obtained from the calibration curve of Figure 7b, plotted with the secondary axis (orange).

As can be seen, the plasmon minima shifts to lower wavelengths when temperature increases because the refractive index of the external medium decreases with increasing temperature. In this case, the minimum is displaced by about 13 nm with a temperature variation of 30 °C, which leads to a change of 0.4213 nm/°C.

We can also estimate the variation of the refractive index of the medium when the temperature changes from the slope of the experimental data by using the calibration curve of the sensor (the sensitivity of this sensor is 2014.2 nm/RIU). Dividing the latter by the slope in Figure 8, we obtained a refractive index variation of the order of 2·10^−4^ RIU/°C, whose order of magnitude is appropriate in accordance with the bibliography for the variation of the refractive index with temperature (1·10^−4^ RIU/°C for water and 4·10^−4^ RIU/°C for liquids).

From now on, we will consider, for simplicity, a variation of 1·10^−4^ RIU/°C to obtain the salinity curve in an indirect way with our sensors. The difference between using 2·10^−4^ RIU/°C or 1·10^−4^ RIU/°C is of the order of 0.06% in the worst case, which is, as we can see, really small.

## 5. Salinity Measurements by an SPR Optical Sensor

### 5.1. Salt Concentration Measurements

**Figure 9:**
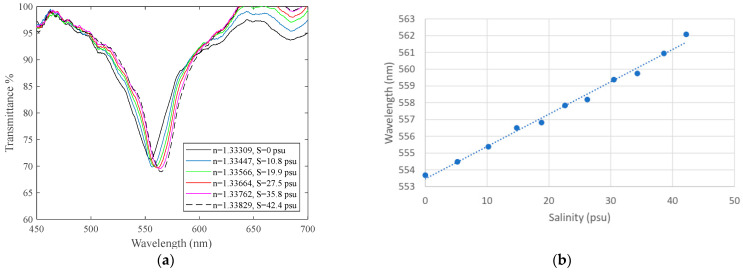
(**a**) Spectral transmittance variations due to outer refractive index changes as KCl concentration varies, with S being the salinity of the external medium measured by a salinometer. (**b**) Spectral transmittance variations due to outer refractive index changes (linear fit: y = 0.1929x + 553.46, R^2^ = 0.9916).

When we have an aqueous external medium in which the salinity value varies, the value of the refractive index will also vary, so the sensor will behave in the same way as when the refractive index of the external medium with ethylene glycol changes, i.e., we will have a figure of minima similar to Figure 7, in which the plasmon moves to longer wavelengths as the salinity increases. Figure 9a shows a representative curve of the spectral transmittance variations due to outer refractive index changes, which, in this case, are associated with variations in KCl concentration in a sample of deionized water. The salinity of the external medium can be measured by a salinometer. In Figure 9b, we plotted the position of the minima for each salinity.

**Figure 10:**
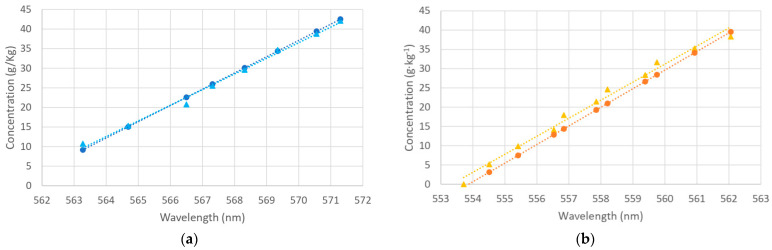
Comparison of the salt concentration for each position of the minimum deduced with the sensor calibration curve with the known sample concentration: (**a**) aquarium salt (blue); (**b**) KCl (orange). Experimental data are represented as circles, and the known concentrations are presented as triangles. Linear fits: y (blue circles) = 4.1513x − 2329.2 (R^2^ = 1); y (blue triangles) = 3.9921x − 2239.0 (R^2^ = 0.9940); y (orange circles) = 4.8308x − 2675.6 (R^2^ = 1); y (orange triangles) = 4.6826x − 2591.1 (R^2^ = 0.9879).

Instead of using a salinometer, we can deduce the salt concentration from the position of the plasmon minima. The refractive index can be obtained from the calibration curve of the sensor in salt concentration, and by means of the relationship between the salt concentration and the refractive index shown in Figure 4, the salt concentration can be calculated. Since the calibration curve with the salt concentration depends on the kind of salt present in the sample, and this is a priori unknown, we have used the calibration curve with ethylene glycol and evaluated whether the results are adequate. To check the goodness of the value of salt concentration obtained with our sensor using this method, we compare these deduced salt concentrations with the known salt concentrations of the samples prepared. For this purpose, the external medium is prepared from a fixed volume of a mixture of deionized water with a salt of known concentration, to which small volumes of higher salinity are added. The results can be seen in Figure 10 for two different types of salt (KCl and aquarium salt).

It can be seen that the curves in both cases are almost coincident, so our method for estimating salt concentration works quite well, with no need to use any algorithm. From the slope of the curves in Figure 10, the sensitivity in the concentration measurement can be derived, around 0.24 nm/(g·kg^−1^) in the case of aquarium salt and 0.21 nm/(g·kg^−1^) for KCl.

**Figure 11:**
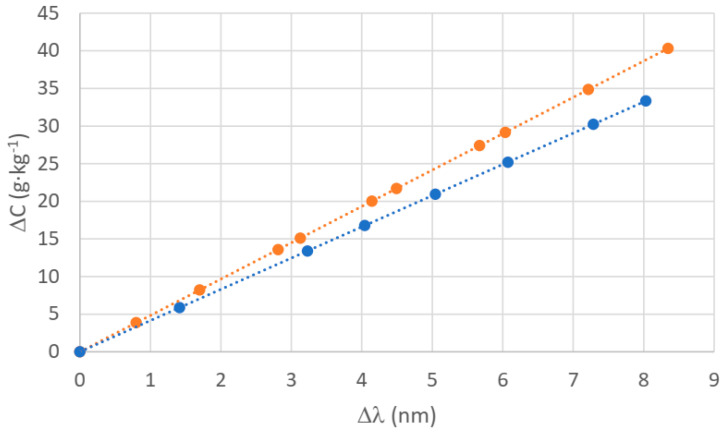
Wavelength shifts for a given salt concentration increment: aquarium salt (blue; linear fit: y = 4.1513x + 6·10^−13^, R^2^ = 1); KCl (orange; linear fit: y = 4.8308x + 7·10^−13^, R^2^ = 1).

The results are slightly different depending on the type of salt, and it fits a little better with the aquarium salt. For a better comparison of the performance depending on the type of salt, in Figure 11, we show the wavelength shifts for a given salt concentration increment for both KCl and aquarium salt, taking the values shown in Figure 10. It can be seen that the effect is less pronounced in the case of aquarium salt than for KCl. In any case, we can see that the sensitivity of the measurements for any kind of salt is high.

### 5.2. Salinity vs. Refractive Index: Comparison between Experimental Measurements and the Use of Algorithms

The characterization of our devices shows that once we have a specific concentration, the location of the minima of the plasmon curve could experimentally provide the value of the salinity of the mixture. Of course, the position of those plasmonic minima corresponds to values of refractive index, which is the parameter our sensor directly measured, being a refractometer. The relationship between the plasmon wavelengths and the refractive index is known to us independently, having performed a general characterization of the sensor with ethylene glycol, as previously discussed.

Traditionally, the relationship between the refractive index of seawater and salinity (plus temperature and pressure, associated with depth), which could be a rather complex one, has been established via semi-empirical algorithms obtained from samples collected in different oceans and seas. Among them, the most widely used is that of Quan and Fry, which is valid for a wavelength range between 400 and 700 nm, although it is commonly used also outside this range [40]. Nowadays, in most papers dealing with salinity measuring devices, this is the algorithm currently used.

A comparison was then conducted of the experimental relationship between the salinity we prepared for the characterization and its refractive index using two different procedures. First, we have the results depicted in Section 5.1. Second, we can use the values of the refractive index obtained with our measurements, introduce them in the Quan and Fry algorithms, and obtain a “calculated” equivalent salinity in this way.

**Figure 12:**
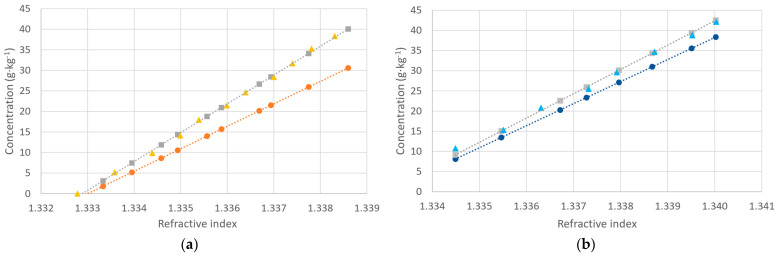
Relationship between the concentration and the refractive index for KCl (**a**) and aquarium salt (**b**) in three cases: direct measurement by the Abbe refractometer (triangles), direct measurement by our sensor (squares) and calculation by the algorithm of Quan and Fry (circles). Linear fits: y (orange circles) = 5467.5x − 7288.2 (R^2^ = 1); y (orange triangles) = 7013.8x − 9348.6 (R^2^ = 0.9983); y (grey squares KCl) = 7013.8x − 9348.6 (R^2^ = 1); y (blue circles) = 5467.5x − 7288.2 (R^2^ = 1); y (blue triangles) = 5724.4x − 7629.1 (R^2^ = 0.998); y (grey squares aquarium salt) = 6027.3x − 8034.2 (R^2^ = 1).

In Figure 12, we show three different curves for two different kinds of salts (KCl and aquarium salt). For the three curves, we show the value of the refractive index of the mixture on the *x*-axis and the concentration that can be obtained from the measurements on the *y*-axis. The three curves are the following: (1) a curve of the refractive index measured by the Abbe refractometer for each mixture (triangles), (2) a curve of the refractive index measured by our sensor (squares), and (3) a curve obtained by using the Quan and Fry algorithm (circles). To obtain curve 2, we took the values obtained directly from the plasmon curves shown in Figure 10 and transformed the wavelengths in the equivalent refractive indices using the experimental relationship between salinity and the refractive index determined by the characterization with the Abbe refractometer in Figure 5. To obtain curve 3, we use these refractive indices to obtain the concentration in the Quan–Fry formula.

We can see that the agreement between our sensor and the Abbe refractometer is very good, regardless of the type of salt used. The behavior of the algorithm of Quan and Fry is better in the case of the aquarium salt, as could be expected since the algorithm was developed for seawater and not for KCl. In any case, it is clear we do not need to rely on an empirical algorithm for the “translation” of the refractive index salinity readings any longer, as the salinity is directly related to the concentration, and given our experimental curves, we have enough data. At the same time, this proves that our results are coherent with those expected if we use the Quan and Fry algorithm. The fact that this algorithm does not work as well with different compounds, as it can be seen with KCl, is another point to consider the use of plasmonic refractometers as the preferred option in case the desire to know or control the salinity of a given solution is not necessarily related to marine research.

### 5.3. Performance According to Measuring Range

In order to obtain a working range extension, we checked the performance of these sensors in near-IR. In near-IR, the DLUWT sensors have proven to have a higher sensitivity [36].

**Figure 13:**
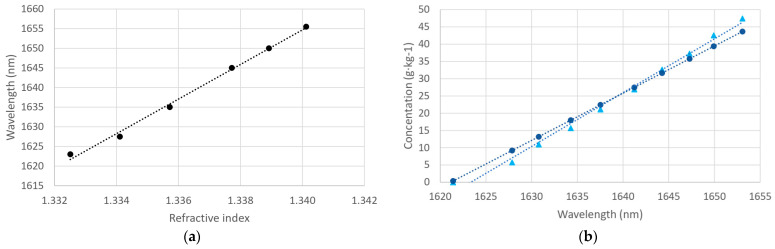
Sensor performance in near IR. (**a**) Calibration curve with ethylene glycol changes (linear fit: y = 4405.9x − 4249.3, R^2^ = 0.995). (**b**) Comparison of the calibration curve in salt concentration with the known sample concentration. Experimental data obtained with the sensor are represented as circle changes (linear fit: y = 1.368x − 2217.7, R^2^ = 1), and the known concentrations are presented as triangles (linear fit: y = 1.558x − 2530, R^2^ = 0.9924).

For that purpose, we calibrated the sensor with ethylene glycol and measured the salt concentration of the external medium fabricated using the aquarium salt in mixtures of different known concentrations. The salt concentration value is obtained, as explained in previous sections. The results are shown in Figure 13.

As expected, the sensitivity in refractive index is higher in near IR than in the visible range (4406 nm/RIU, from Figure 13a). Besides, it can be seen in Figure 13b, where both methods obtained similar salt concentrations, as they occurred in the visible region. From the slope of the curve, the salt concentration sensitivity can be deduced. The sample concentration data indicate it is 0.64 nm/(g·kg^−1^), and the estimation from our sensor is very similar, of 0.73 nm/(g·kg^−1^).

In this sense, in terms of the salt concentration measurement, the improvement is considerable since the sensitivity is three times the sensitivity obtained in the visible region, which was around 0.24 nm/(g·kg^−1^). When using DLUWTs, we can easily change the working spectral range in the design, which allows us to consider it the best option in general for refractometric salinity measurements to shift to higher wavelengths.

## 6. Conclusions

The accurate measurement of salinity is so important that any effort to produce more efficient devices is worth achieving. Two main points were considered here: first, optical measurements and, in particular, refractometric measurements, can and possibly should be preferred to those traditionally based on electrical conductivity, and therefore, the progress in the field of optical salinity sensors is a strategic option that could eventually imply a paradigm shift in the approach to the knowledge and monitoring of salinity, including a new definition of the standards. For that reason, we tried to adequately determine the pros and cons of the optical methods of determining the salinity of seawater. We also comparatively characterized the different instruments available based on varied physical principles.

Then, once it was decided that refractometry is a good way of approaching the problem, we presented a plasmonic device, taking into account that, within the field of refractometers, those based on optical fibers present evident advantages, as discussed in detail in the paper. In addition, within the field of fiber optic sensors, those based on surface plasmon resonance (SPR) perform very well in terms of sensitivity. In our case, the specific structure that employed doubly deposited uniform-waist tapered optical fibers (DLUWTs) incorporated versatility into their design, allowing for different wavelength regions to be explored, showing that the step towards the infrared communication range permits increased sensitivity while maintaining all the good characteristics of the devices.

In the experimental results provided here, we can see how this shift towards a new concept of salinity, based on an optical standard and determined via plasmonic refractometry, is no longer only a desirable possibility but a reality. There is an understandable inertia when a technique is already established. In this case, well-tested and widely extended electrical conductivity-based measurements have seen significant changes from an operative, day-to-day point of view in the last two or three decades, where the feasibility of in situ, real-time measurements of salinity with optical sensors have been successfully demonstrated.

It is our opinion that the variety of results present in the literature and the progressive implementation of physical principles, techniques, and devices have proven their optimum performance in chemical sensors. In addition, the substitution of the measuring elements in environmental monitoring plants or in industries related to the exploitation of marine resources, as well as in systems employed for better knowledge of climate dynamics, will become more common. Our group, which was among the pioneers in using fiber optic plasmonic sensors in the in situ measurements of salinity in oceanographic campaigns, would like to contribute to this change with proposals like the one presented here.

## Data Availability

Data are contained within the article.
